# Behavioral tests of auditory processing: a study on reference values for normal-hearing adults

**DOI:** 10.1590/2317-1782/e20250151en

**Published:** 2026-04-02

**Authors:** Sthella Zanchetta, Laura Caetano Meneghelli, Pamela Papile Lunardelo

**Affiliations:** 1 Departamento de Ciências da Saúde, Faculdade de Medicina de Ribeirão Preto, Universidade de São Paulo – FMRP-USP - Ribeirão Preto (SP), Brasil.; 2 Faculdade de Medicina de Ribeirão Preto, Universidade de São Paulo – FMRP-USP - Ribeirão Preto (SP), Brasil.; 3 Departamento de Formação Específica em Fonoaudiologia, Universidade Federal Fluminense – UFF - Nova Friburgo (RJ), Brasil.

**Keywords:** Auditory Perception, Auditory Tests, Adults, Auditory Disorder, Audiology

## Abstract

**Purpose:**

To analyze and compare outcomes of clinical tests assessing auditory processing (AP) in adults with normal hearing across age groups and to propose reference values.

**Methods:**

A total of 116 adults aged 18 to 59 years, all with normal hearing, no auditory complaints, and native speakers of Brazilian Portuguese, were evaluated. The following verbal and nonverbal tests were administered: Masking Level Difference (MLD), Gaps-In-Noise (GIN), Pitch Pattern Sequence (PPS), Duration Pattern Sequence (DPS), and Dichotic Digits Test (DDT).

**Results:**

The GIN, PPS, and DDT showed significant differences beginning at 40 years of age. For the DPS, significant differences were observed between individuals aged 18–29 years and those aged 50–59 years. In the DDT, significant differences were found in the right ear beginning at 50 years and in the left ear beginning at 40 years. The MLD was the only test that did not show age-related differences.

**Conclusion:**

A decline in auditory processing test performance was observed with increasing age, even before senescence. The age at which significant changes occurred varied across tests, highlighting the need to establish age-specific reference values. The MLD was the only test that remained stable throughout adulthood. The proposed reference values are as follows: MLD, 8–14 dB (18–59 years); GIN, ≤ 6 ms (18–39 years) and ≤ 8 ms (40–59 years); PPS, ≥ 76.6% (18–39 years) and ≥ 63.0% (40–59 years); DPS, ≥ 90.3% (18–29 years), ≥ 80.0% (30–49 years), and ≥ 70.0% (50–59 years); DDT–RE, ≥ 98.5% (18–49 years) and ≥ 93.2% (50–59 years); DDT–LE, ≥ 97.5% (18–39 years) and ≥ 95.0% (40–59 years).

## INTRODUCTION

There is consensus that Auditory Processing Disorder (APD) involves an impairment in the perception and processing of auditory information, both verbal and nonverbal, within the central auditory nervous system (CANS), affecting children, adults, and older individuals^(1-3)^.

Scientific organizations describe the indications for auditory processing (AP) assessment in adults, including difficulties performing auditory tasks related to work or leisure activities (e.g., music), learning a new language or achieving proficiency in it, neurological conditions of any etiology, cognitive decline, tumor masses, or a “disproportionate” complaint of listening difficulties in noisy environments^(1,4,5)^. Another specific indication includes individuals who reach adulthood with preexisting APD, such as those with neurodevelopmental disorders, including autism spectrum disorder^(2,6)^.

Assessment for identifying APD should be conducted using a battery of behavioral tests that includes both verbal and nonverbal stimuli^(1-3)^. Although there is no recommendation for a “minimum test battery,” there is agreement that selected tests should assess different auditory mechanisms or categories reflecting auditory skills, such as speech perception under reduced redundancy, verbal dichotic listening, temporal processing mechanisms (resolution, ordering, and masking), binaural interaction, and sound localization^(7-9)^.

Despite this knowledge, studies addressing APD in adults remain limited. A systematic review found that some studies focused on characterizing specific auditory mechanisms or skills within particular diseases (e.g., diabetes), rather than on identifying APD itself^(10)^.

Adulthood spans the age range from 18 to 59 years, and evidence indicates that performance on behavioral auditory tests declines with increasing age within this life stage^(11-21)^. A study investigating temporal resolution tests^(1,5)^ demonstrated that adults older than 31 years performed worse on the Gaps-In-Noise (GIN) test than younger adults, whereas significant differences in the Random Gap Detection Test (RGDT) emerged only after 41 years of age. Another study^(21)^ reported a decline in performance on both verbal and nonverbal tests comprising the clinical AP assessment across adulthood. The authors reported, for example, that adults aged 40 years and older performed worse than those aged 18-39 years on the Dichotic Digits Test (DDT) and the GIN test, whereas significant differences on the Duration Pattern Test (DPT) occurred only from 50 years of age onward. For the dichotic sentence identification test^(14)^, significant differences were observed between adults aged 20–29 years and those aged 30-39 years. These age-related changes in auditory test performance during adulthood, observed in “eutrophic” individuals that is, in the absence of comorbidities, have been described as “healthy decline” and are thought to reflect natural changes in the CANS^(21)^.

Performance changes associated with increasing age, even during the pre-senescent period, underscore the challenge of identifying APD in adulthood in the absence of age-specific normative values. Reference values have been proposed for some tests, and these studies have made important contributions to AP assessment^(14,15,19,22-25)^. However, additional information is still needed, particularly regarding the ages at which performance changes emerge. One study^(19)^ evaluated adults aged 18–58 years using several AP tests and divided participants into two age groups: 18–29 years and 30–58 years. This latter group is broad and includes ages at which significant performance differences have already been reported for some tests^(14,15,21)^. Other authors evaluated a wider age range but focused exclusively on the GIN and RGDT tests^(15)^, or proposed reference values only for individuals aged 35 years or younger^(24)^.

Based on evidence documenting performance changes in adults, the recommendation to use a battery of tests for APD identification, and gaps in reference values across decades of adult life for AP tests, the present study was designed to test the hypothesis that test results differ as a function of increasing age. To examine this hypothesis, we evaluated adults with normal hearing aged 18 to 59 years using different AP tests, with the aim of comparing performance across age groups and proposing reference values to support diagnostic assessment of APD.

## METHODS

This observational, cross-sectional study was approved by the Institutional Research Ethics Committee under protocol numbers 2,816,793 and 4,826,213. All participants provided written informed consent prior to participation.

### Participants

Individuals aged 18 to 59 years, with normal hearing and good general health, were eligible for inclusion. Convenience sampling was adopted. Participants were recruited through personal contact by researchers, announcements on institutional social media platforms, and regional radio and television programs. The recruitment message stated: *“If you have no hearing complaints and are in good general health, how about having your hearing evaluated?”* Recruitment and assessments were conducted in 2019 and again between 2021 and 2025.

During initial contact with a researcher by telephone, text message, or email, screening questions were used to confirm eligibility and identify additional inclusion criteria, namely: (a) absence of hearing loss, either previously documented or self-reported; (b) completion of at least secondary education; (c) good general health, defined as the absence of metabolic, autoimmune, or neurological diseases, as well as prior neurological events such as stroke or traumatic brain injury; (d) no history of otologic surgery; and (e) no occupational exposure to high sound pressure levels.

Exclusion criteria, verified on the day of data collection, included identification of hearing loss of any type in at least one ear and an altered score on the Mini-Mental State Examination (MMSE).

A total of 122 individuals attended the assessment. Six individuals were excluded: one (0.8%) due to an altered MMSE score and five (4.1%) due to hearing loss. These individuals received feedback regarding their assessments and were referred for specialized evaluation and management. AP tests were ultimately performed in 116 participants.

### Procedures

All auditory tests were conducted in a sound-treated booth using an Astera 2 audiometer (Interacoustics) with HDA 300 headphones. Acoustic immittance measures were obtained using a Zodiac immittance meter (Madsen) with a 226-Hz probe tone.

Initial procedures included: (a) otoscopy; (b) pure-tone audiometry; (c) tympanometry; and (d) acoustic reflex testing.

Pure-tone audiometry was performed at frequencies ranging from 0.25 to 8 kHz. Normal hearing was defined as a pure-tone average of thresholds at 0.5, 1, 2, and 4 kHz ≤ 20 dB HL^(26)^, and a speech reception threshold between 0 and 10 dB SL relative to the corresponding pure-tone average. Tympanometry and acoustic reflex testing were considered complementary assessments. Tympanometric curves of type A, Ar, or Ad^(27)^ were considered normal when accompanied by the presence of contralateral acoustic reflexes at 0.5, 1, and 2 kHz.

The MMSE was administered by one of the authors to all participants. Only individuals with scores of ≥ 29 proceeded to AP testing^(28)^.

### Auditory processing tests

All tests were administered at an intensity of 50 dB SL, referenced to the speech pure-tone threshold of each ear, or at the level of greatest listening comfort reported by the participant.

### Masking Level Difference (MLD)^(29)^

The MLD test consists of 33 narrowband noise segments, some of which contain five pulsed tones. The test includes two variables: the tone–noise relationship, which alternates across segments, and the phase of the tones (SoNo and SπNo). There are seven segments without pulsed tones, ten segments with pulsed tones in the same phase as the noise (SoNo), and 12 segments with pulsed tones in opposite phase to the noise (SπNo). The MLD result was calculated as the difference, in decibels, between the SπNo and SoNo conditions.

### Gaps in Noise (GIN)^(30)^

The GIN test consists of a series of noise segments (ranging from 29 to 36, depending on the test list used), each lasting 6 s, with 5-s intervals between segments. Each segment may contain up to three silent intervals, or none. When present, silent intervals have durations of 2, 3, 4, 5, 6, 8, 10, 12, 15, and 20 ms. Each gap duration occurs six times across all lists, with randomized distribution. The test was administered monaurally using test track 3, and the order of ear testing was alternated across participants. The approximate gap detection threshold (A-GDT) was calculated according to the test author’s recommendations.

### Temporal ordering tests

The Pitch Pattern Sequence (PPS) and Duration Pattern Sequence (DPS) tests (adult version, Auditec)^(31)^ were administered binaurally. Performance was calculated as the percentage of correct responses for each test.

The PPS is composed of two pure tones of equal duration (200 ms) but different frequencies: a low-frequency tone at 880 Hz and a high-frequency tone at 1430 Hz. Stimuli are organized into 30 sequences, each comprising three tones. Each sequence contains two tones of the same frequency and one tone of a different frequency, arranged in six possible patterns: HHL, HLH, HLL, LLH, LHL, and LHH. Interstimulus intervals were 150 ms, and intersequence intervals were 7 s. Participants were instructed to verbally label the tones according to pitch, in the order presented.

The DPS is composed of two pure tones of the same frequency (1000 Hz) but different durations: a short tone (250 ms) and a long tone (500 ms). As in the PPS, the test includes 30 three-tone sequences arranged in six possible patterns: SSL, SLL, SLS, LLS, LSS, and LSL. Interstimulus intervals were 300 ms, and intersequence intervals were 6 s. Participants were instructed to verbally identify the patterns using the terms “short” and “long,” in the order heard.

### Dichotic Digits Test (DDT)^(32)^

The Brazilian Portuguese version of the DDT consists of 20 sequences, each comprising four digits. Two digits are presented simultaneously to the right and left ears, followed by a second pair presented dichotically. The digits used are four, five, seven, eight, and nine, combined such that no digit is repeated within a sequence. The test was administered under the binaural integration condition, and scoring followed procedures specified in the test manual^(32)^.

### Data analysis

Analyses were performed to characterize the sample according to sociodemographic variables and to examine whether AP test performance was associated with age. When an association was identified, additional analyses were conducted to determine which age groups differed in performance.

Sex distribution and years of education across age groups were analyzed using the chi-square test and the Kruskal–Wallis test, respectively. The relationship between AP test performance and age was examined in two stages. First, the presence or absence of a correlation was assessed using Spearman’s correlation test. Second, test performance was compared across age groups using the Kruskal–Wallis test when more than two age groups were included. When significant differences were observed, Dunn’s post hoc test was applied to identify which groups differed. The Mann–Whitney test was used when comparisons involved only two age groups and was also applied to compare GIN results between ears.

To evaluate the feasibility of proposing reference values for each AP test, means, standard deviations, and percentiles were calculated. The significance level was set at 5%, and statistically significant results were indicated by an asterisk (*).

## RESULTS

AP tests were administered to 116 participants. For sample characterization by age, sex, and years of education, participants were stratified into four age groups (Table 1). Sex distribution and years of education did not differ significantly across age groups (p > 0.05).

**Table 1:** Distribution and characterization of the variables age, sex, and education level among age groups

Variables	Measurements	Age group				*p-value*
		18-29	30-39	40-49	50-59	
		N=30	N=29	N=29	N=28	
Age	M±sd	22.4±2.6	33.4±2.5	44.6±3.2	53.8±1.3	
Sex (fem.)	n/%	16/53.3	15/51.7	17/58.6	17/60.7	0.8883#
Education level (years)	M±sd	15.3±2.7	17.1±3.5	16.4±2.7	15.5±4.1	0.0601##
	Min-max	12-18	12-22	11-23	11-23	

# Chi-square test;

## Kruskal-Wallis Test;

**Caption:** fem = female; M = mean; sd = standard deviation

For the subset of tests administered to 20 participants (MLD and GIN), sex distribution was balanced, with 50.0% (10/20) female participants in each age group. Likewise, no significant differences in years of education were observed within this subset (p = 0.0732; 14.3 ± 1.7, 18.1 ± 2.2, 15.8 ± 2.9, and 15.3 ± 3.0 years for groups 1–4, respectively).

Variation in the number of participants across tests reflects the methodological design of the study, which integrated data from two cross-sectional cohorts conducted by the same research team and aligned with identical inclusion criteria. Not all tests were administered in both cohorts because of differences in research objectives. Nevertheless, all tests followed standardized protocols, ensuring consistency in assessment procedures.

The results of AP tests by age group are presented in Table 2.

**Table 2:** Scores by age group

Tests	Measures	Age group			
		18-29	30-39	40-49	50-59
MLD (dB)	n	20	20	20	20
	M ± dp	10.8 ± 1.8	10.8 ± 2.1	10.9 ± 2.1	11.1 ± 2.1
	Median	10	10.0	10	12
	Min-max	6-14	8-16	4-14	8-16
GIN-RE (ms)	n	20	20	20	20
	M ± sd	5.4 ± 0.9	5.0 ± 0.9	6.5 ± 1.1	7.4 ± 1.1
	Median	5.5	5.0	6.0	8.0
	Min-max	4.0-8.0	3.0-6.0	5.0-8.0	5.0-10.0
GIN-LE (ms)	n	20	20	20	20
	M ± sd	5.4 ± 0.9	4.9 ± 0.9	6.5 ± 1.1	7.1 ± 1.2
	Median	5.0	5.0	6.0	8.0
	Min-max	4.0-8.0	3.0-6.0	5.0-8.0	5.0-10.0
PPS (%)	n	30	29	29	28
	M ± sd	94.8 ± 7.8	92.5 ± 9.3	86.6 ± 12.8	83.6 ±13.2
	Median	100.0	100.0	86.6	86.6
	Min-max	73.3-100.0	73.3-100.0	60.0-100.0	46.6-100.0
DPS (%)	n	20	20	20	20
	M ± sd	96.3 ± 6.3	91.9 ± 9.7	90.8 ± 10.15	86.1 ± 12.9
	Median	100.0	93.3	93.3	90.0
	Min-max	73.3-100.0	60.0-100.0	63.3-100.0	53.3-100.0
DDT-RE (%)	n	30	29	29	28
	M ± sd	99.9±0.3	99.4±0.9	98.9±2.0	97.7±2.1
	Median	100.0	100.0	100.0	98.7
	Min-max	98.7-100.0	96.2-100.0	91.2-100.0	88.7-100.0
DDT-LE (%)	n	30	29	29	28
	M ± sd	99.2±1.7	99.45±0.8	98.2±1.9	98.4±1.7
	Median	100.0	100.0	98.7	98.7
	Min-max	98.7-100.0	97.5-100.0	92.5-100.0	93.7-100.0

**Caption:** ms = milliseconds; M = mean; sd = standard deviation; Min = minimum value; Max = maximum value; % = percentage; n = number of participants; MLD = Masking Level Difference; GIN = Gaps-In-Noise; PPS = Pitch Pattern Sequence; DPS = Duration Pattern Sequence; DDT-RE = Dichotic Digit Test right ear; DDT-LE = Dichotic Digit Test left ear

Because the GIN test was administered monaurally, the initial analysis compared ear-specific results within each age group. No statistically significant differences were observed between ears in any age group (p > 0.05): 18–29 years (p > 0.999), 30–39 years (p = 0.5000), 40–49 years (p > 0.9999), and 50–59 years (p = 0.2500). These findings support the analysis of GIN performance based on the total number of ears rather than the number of participants.

### Test results as a function of age

**Figure 1:**
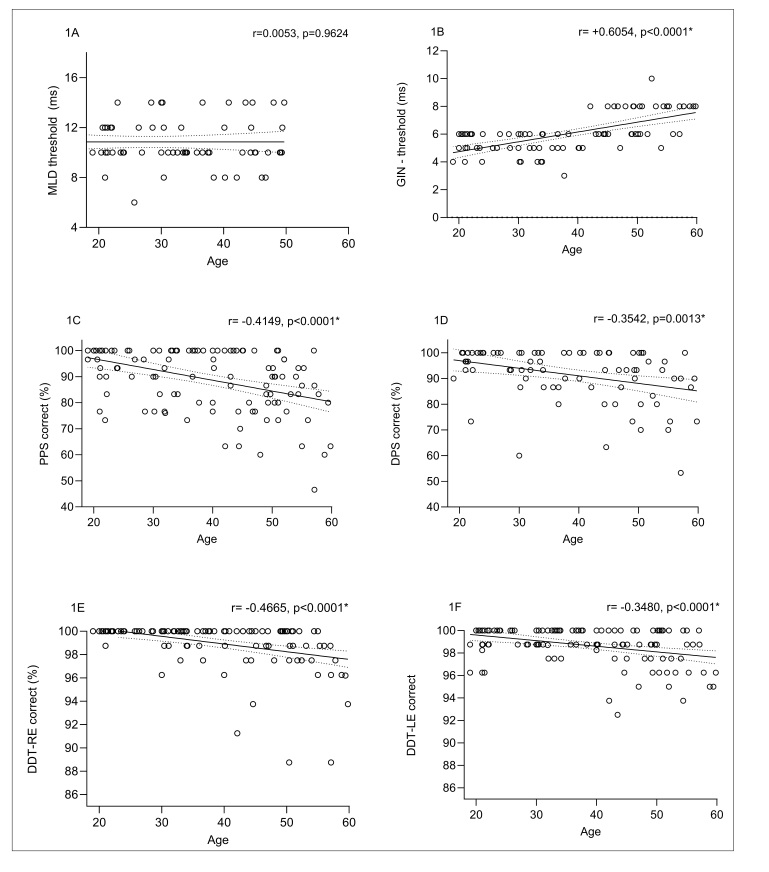
Correlation between performance on the MLD (A), GIN (B), PPS (C), DPS (D), and DDT (E and F) tests as a function of age

Analyses were performed to examine whether AP test performance was significantly correlated with increasing age, considering all 116 participants without stratification by age group (Figure 1). All tests, except the MLD (p > 0.05), showed statistically significant correlations with age (p < 0.05)*. A decline in performance with increasing age was observed for all tests except the MLD.

**Caption:** ms = milliseconds; M = mean; sd = standard deviation; Min = minimum value; Max = maximum value; % = percentage; n = number of participants; MLD = Masking Level Difference; GIN = Gaps-In-Noise; PPS = pitch pattern sequence; DPS = duration pattern sequence; DDT-RE = dichotic digit test right ear; DDT-LE = dichotic digit test left ear

### Analysis for the proposal of reference values

At this stage, AP test performance was compared across age groups. When significant differences were identified, post hoc analyses were conducted to determine which age groups differed. These results were then used to evaluate the need for and feasibility of proposing age-specific reference values for each AP test across adulthood.

**Figure 2:**
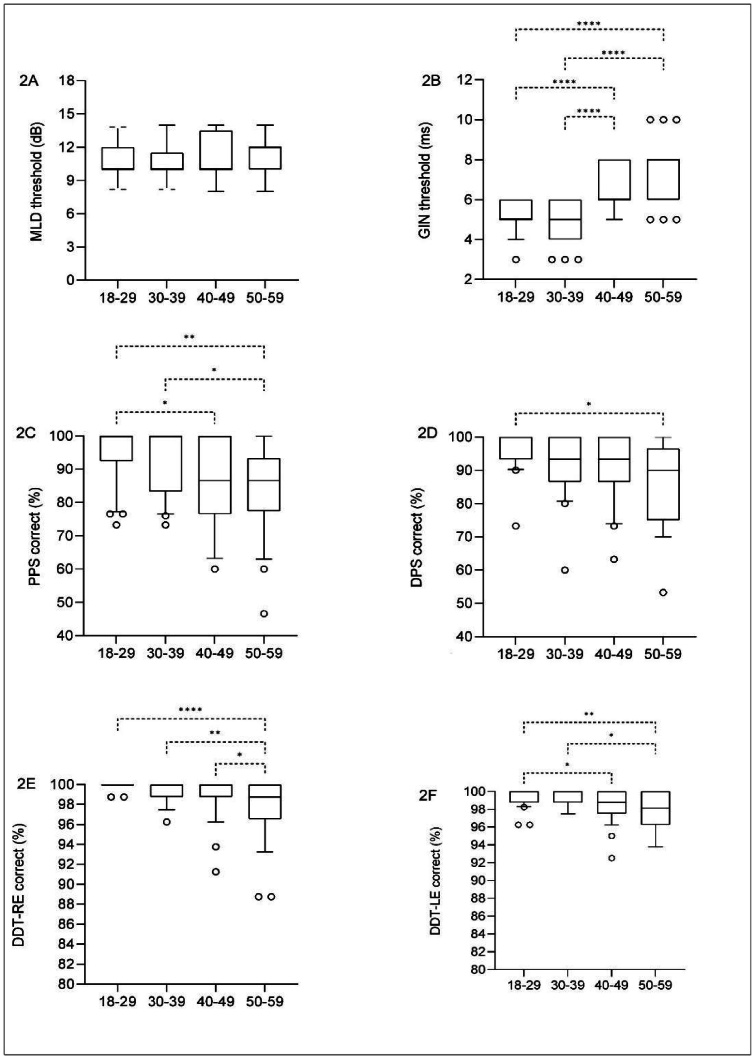
Boxplot illustrating the distribution of performance on the MLD (A), GIN (B), PPS (C), DPS (D), and DDT (E and F) tests across different age groups. The lower and upper bounds of the boxplot represent the 25th and 75th percentiles, the lower and upper whiskers represent the 10th and 90th percentiles, and the internal line represents the median

MLD - No significant differences in MLD thresholds were observed across age groups (p > 0.8352) (Figure 2A). Based on this finding, MLD thresholds were considered stable across the age range of 18–59 years. Mean values, standard deviations, and percentiles are presented in Table 3. Considering the 10th to 90th percentiles, the proposed reference range for typical performance is 8–14 dB (Table 3).

**Caption:** ms = milliseconds; M = mean; sd = standard deviation; Min = minimum value; Max = maximum value; % = percentage; n = number of participants; MLD = Masking Level Difference; GIN = Gaps-In-Noise; PPS = pitch pattern sequence; DPS = duration pattern sequence; DDT-RE = dichotic digit test right ear; DDT-LE = dichotic digit test left ear

**Table 3:** Metrics and proposed performance scores for the MLD, GIN, PPS, DPS, and DDT tests for adulthood

Tests	Age	n	Metrics						
			M ± sd	Percentiles					
				P5	**P10**	P25	P75	**P90**	P95
MLD (dB)									
	18-59	80	10.9 ± 2.0	8	**8**	10	12	14.0	14
GIN (ms)									
	18 – 39	80	5.1 ± 0.8	4	4	5	6	**6**	6
	40 – 59	80	6.5 ± 1.1	5	5	6	8	**8**	8
PPS (%)									
	18 – 39	59	93.7 ± 8.6	76.0	**76.0**	90.0	100.0	**100.0**	100.0
	40 – 59	57	84.7 ± 12.9	60.0	**63.0**	76.6	96.6	**100.0**	100.0
DPS (%)									
	18 – 29	20	96.3 ± 6.3	74.1	**90.3**	93.3	100.0	**100.0**	100.0
	30 – 49	40	91.4 ± 9.5	63.8	**80.0**	86.6	100.0	**100.0**	100.0
	50 – 59	20	86.1 +/- 12.9	54.1	**70.0**	74.9	96.6	**100.0**	100.0
DDT-RE (%)									
	18-49	88	99.4 ± 0.8	97.5	**98.5**	100.0	100.0	**100.0**	100.0
	50 – 59	28	97.6 ± 2.9	88.7	**93.2**	96.5	100.0	**100.0**	100.0
DDT-LE (%)									
	18 – 39	59	99.4 ± 0.9	97.5	**97.5**	98.5	100.0	**100.0**	100.0
	40 – 59	57	97.8 ± 1.9	94.3	**95.0**	96.5	100.0	**100.0**	100.0

**Caption:** ms = milliseconds; M = mean; sd = standard deviation; Min = minimum value; Max = maximum value; % = percentage; n = number of participants; MLD = Masking Level Difference; GIN = Gaps-In-Noise; PPS = pitch pattern sequence; DPS = duration pattern sequence; DDT-RE = dichotic digit test right ear; DDT-LE = dichotic digit test left ear

GIN - Comparison of approximate gap detection thresholds (A-GDT) across the four age groups revealed a statistically significant difference (p < 0.0001*). Post hoc analysis identified significant differences between the 18–29-year group and the 40–49-year (p = 0.0001*) and 50–59-year groups (p < 0.0001*), as well as between the 30–39-year group and the 40–49-year (p < 0.0001*) and 50–59-year groups (p < 0.0001*). No significant differences were observed between the 18–29- and 30–39-year groups (p > 0.9999), nor between the 40–49- and 50–59-year groups (p = 0.2176) (Figure 2B). The older age groups exhibited higher A-GDT values, indicating poorer temporal resolution performance.

Based on these findings, results were regrouped into two age ranges: 18–39 years and 40–59 years. Subsequent comparison between these age ranges revealed a statistically significant difference (p < 0.0001*). Descriptive statistics, including means, standard deviations, and percentiles, are presented in Table 3. Using the 90th percentile, an A-GDT of ≤ 6 ms is proposed as the reference value for individuals aged 18–39 years, and ≤ 8 ms for those aged 40–59 years (Table 3).

PPS - Comparison of PPS accuracy across the four age groups revealed a statistically significant difference (p = 0.0005*). Post hoc analysis identified significant differences between the 18–29-year group and the 40–49-year (p = 0.0273*) and 50–59-year (p = 0.0015*) groups, as well as between the 30–39-year and 50–59-year groups (p = 0.0240*). No significant differences were observed for the remaining comparisons (18–29 vs. 30–39, p > 0.9999; 30–39 vs. 40–49, p > 0.2401; 40–49 vs. 50–59, p > 0.9999) (Figure 2C). Older participants exhibited poorer performance.

Based on these findings, PPS scores were regrouped into two age ranges: 18–39 years and 40–59 years. Comparison between these age ranges demonstrated a statistically significant difference (p < 0.0001*). Descriptive statistics are presented in Table 3. Using the 10th percentile, reference values of ≥ 76.6% for individuals aged 18–39 years and ≥ 63.3% for those aged 40–59 years are proposed.

DPS - A statistically significant difference in DPS performance was observed across age groups (p = 0.0196*). Post hoc analysis identified that this difference occurred only between the 18–29-year and 50–59-year groups (p = 0.0112*), with no significant differences among the remaining comparisons (18–29 vs. 30–39, p = 0.5622; 30–39 vs. 40–49, p > 0.9999; 30–39 vs. 50–59, p = 0.9082; 40–49 vs. 50–59, p > 0.9999) (Figure 2D). These findings indicate that only the youngest and oldest age groups differed significantly, with poorer performance observed in the oldest group (Table 3).

Based on these findings, three age ranges were defined for reference values: 18–29, 30–49, and 50–59 years. Comparative analysis across these three age ranges again revealed a statistically significant difference (p = 0.0074*), with the only significant contrast observed between the youngest and oldest groups (p = 0.0056*), and no differences among the remaining comparisons (p > 0.05). Descriptive statistics are presented in Table 3. Using the 10th percentile, the proposed reference values are ≥ 90.3% for individuals aged 18–29 years, ≥ 80.0% for those aged 30–49 years, and ≥ 70.0% for those aged 50–59 years (Table 3).

DDT - Right ear (RE) and left ear (LE) scores were analyzed separately. Statistically significant differences were observed across age groups for both ears (RE, p < 0.0001*; LE, p = 0.0097*).

For RE performance, post hoc analysis identified significant differences between the 18–29, 30–39, and 40–49-year groups compared with the 50–59-year group (p < 0.0001*, p = 0.0029*, and p = 0.0365*, respectively), with the 50–59-year group exhibiting lower scores (Figure 2E). No significant differences were observed for the remaining comparisons (18–29 vs. 30–39, p = 0.6770; 18–29 vs. 40–49, p = 0.1131; 30–39 vs. 40–49, p > 0.999).

Based on these findings, reference values for the RE were analyzed using two age ranges: 18–39 years and 40–59 years. Comparison between these age ranges revealed a statistically significant difference (p < 0.0001*). Descriptive statistics are presented in Table 3. Using the 10th percentile, the proposed reference values are ≥ 98.5% for individuals aged 18–39 years and ≥ 93.2% for those aged 40–59 years (Table 3).

For LE performance, comparison across the four age groups revealed a statistically significant difference (p = 0.0010*). Post hoc analysis identified significant differences between the 18–29-year group and the 40–49- and 50–59-year groups (p = 0.0499* and p = 0.0196*, respectively), as well as between the 30–39-year group and the 40–49- and 50–59-year groups (p = 0.0442* and p = 0.0173*, respectively) (Figure 2F). No significant differences were observed between the 18–29- and 30–39-year groups (p > 0.9999), nor between the 40–49- and 50–59-year groups (p > 0.9999).

LE results were regrouped into two age ranges: 18–39 years and 40–59 years. Comparative analysis between these age ranges showed a statistically significant difference (p = 0.0063*). Descriptive statistics are presented in Table 3. Using the 10th percentile, the proposed reference values are ≥ 97.5% for individuals aged 18–39 years and ≥ 95.0% for those aged 40–59 years.

## DISCUSSION

The present study aimed to investigate and compare the results of the MLD, GIN, PPS, DPS, and DDT tests in normal-hearing adults as a function of age and to propose reference values.

Participant recruitment was designed to ensure community representativeness. The preservation of peripheral hearing sensitivity, the absence of impairment in mental status, and homogeneity in educational level minimized the influence of variables known to affect performance on AP tests^(21)^. This methodological approach strengthens the interpretation that the performance differences observed in the present study are primarily attributable to age, thereby reducing the likelihood of confounding effects.

The use of percentiles to define reference values warrants further consideration. This metric has been widely used in the interpretation of AP tests^(14,33-36)^, although the use of the mean plus two standard deviations has been more commonly recommended^(1-3,37-39)^.

In the present study, auditory test results did not follow a normal distribution. Consequently, mean accuracy does not adequately represent the most frequent response pattern, and measures of dispersion become less informative. Under these circumstances, the use of the standard deviation or standard error does not accurately reflect variability, making percentiles the more appropriate metric^(40)^.

Percentiles allow an ordered distribution of responses and extend beyond a binary classification (normal vs. abnormal), positioning an individual relative to their reference group^(40)^. The combined use of measures of central tendency and percentiles has previously been adopted in studies proposing normative values for AP tests. In one such study, minimal differences were observed between the two approaches, leading the authors to recommend the use of central tendency measures^(41)^. Other studies have also reported more than one metric, such as the mean plus two standard deviations and percentiles, to describe normative data^(35,36)^. In the present study, with the exception of the DPS, the 10th and 90th percentiles for the MLD, GIN, PPS, and DDT were closely aligned with the values derived from the mean plus two standard deviations. Nevertheless, the reference values proposed here were derived from the 10th and 90th percentiles. Additional percentiles were included to allow a more precise characterization of performance, both for diagnostic assessment and for post–auditory training follow-up^(40)^.

### Reference values for AP tests

Structural and physiological changes in the CANS during adulthood significantly influence performance on clinical AP tests, with the exception of the MLD. These changes may be attributed to neurophysiological alterations and to modifications in neurotransmitter systems within the central nervous system^(11,20,21,42)^. The age at which these changes became evident varied across tests and, in some cases, between ears, as observed for the DDT. This pattern is expected, given that physiological and biochemical changes occur at different times depending on the brain region involved^(11,20)^.

The MLD was the only test that did not show significant variation in performance across age groups, allowing a single reference threshold to be established for adults aged 18 to 59 years. The MLD assesses the binaural interaction mechanism, which is related to auditory function at the level of the eighth cranial nerve and the brainstem (i.e., subcortical structures)^(43)^. Therefore, the absence of age-related differences within this age range may be explained by the fact that, up to approximately 60 years of age, age-related changes predominantly affect cortical regions. The reference range of 8 to 14 dB, based on the 10th and 90th percentiles, is close to values previously reported (10.2 to 11.4 dB) in women aged 20 to 30 years^(2,5)^. However, these values are lower than the 17–18 dB thresholds suggested by another study, which included individuals aged 18 to 58 years^(19)^.

For the GIN test, no differences between ears were observed across age groups, consistent with previous reports^(15,23)^. A decline in approximate gap detection thresholds from 40 years of age onward has been documented in a study evaluating 176 individuals aged 20 to 85 years, in which the authors attributed this finding to age-related changes in the CANS^(13)^. In contrast, another study did not identify differences in gap detection thresholds between young and middle-aged adults; however, a reduced percentage of gap identification was observed in the older group, a finding associated with decreased temporal processing efficiency in middle-aged women^(44)^.

The reference values for GIN thresholds, derived from the 90th percentile, were 6 ms for individuals aged 18–39 years and 8 ms for those aged 40–59 years, which are identical to previously suggested values^(15)^. These thresholds are also comparable to those reported in other studies, such as 4.19 ms for young adults^(23)^, 6 ms for adults with a mean age of 39 years^(36)^, and 6.6 ms (±1.3) for middle-aged adults^(45)^.

For the PPS, reference values derived from the 10th percentile were 76.6% for individuals aged 18–39 years and 63.3% for those aged 40–59 years. Another study proposed values ≥ 86.6% for individuals aged 18–29 and 30–58 years^(19)^, using the mean plus two standard deviations. Among Spanish-speaking adults, reference values ≥ 71.4% were proposed for individuals aged 18 to 50 years, with a mean age of 29 years^(35)^, values that are closer to those reported in the present study.

Other studies have employed a different version of the PPS^(46)^, which differs in tone frequency (1122 Hz vs. 1430 Hz), tone duration (150 ms vs. 200 ms), and intertone interval (200 ms vs. 150 ms) compared with the version used in the present study. In a study of 28 Dutch adults aged 18 to 47 years (mean age 29 years), a reference value of 89% correct responses (10th percentile) was reported^(33)^. In another study involving 76 Polish adults aged 18 to 54 years (mean age 39 years), a reference value of 56.7% (10th percentile) was proposed^(36)^. These discrepancies indicate that PPS reference values are not homogeneous, even when the same test version is applied. Factors such as small sample sizes, broad age ranges, and native language may contribute to these differences, with language being particularly relevant for temporal ordering tests^(47)^.

For the DPS, three age groups with distinct normative values were established. The cutoff value of 90.3% (10th percentile) for individuals aged 18–29 years is higher than the 73.3% previously reported^(19)^. For individuals aged 30–49 years, the proposed value was 80.0%, also higher than the 70% previously suggested for this age range^(19)^. Another study reported 83.3% (3rd percentile) for young Brazilian adults using a different version of the DPS^(46)^, with the only difference being the intersequence interval (7 vs. 6 s)^(22)^. In that study, the 25th percentile value of 93.3% was also reported, which coincides with the value observed in the present study for the same percentile. Studies conducted in speakers of other languages have demonstrated wide variability. In Polish adults aged 18 to 54 years (mean age 39 years), a reference value of 55.3% (10th percentile) was proposed^(36)^. The version of the DPS used in the present study recommends a normative cutoff of 67%^(31)^, whereas another version suggests 73% (10th percentile)^(46)^. Other studies have reported mean scores of 86.5% (±13.0) and 79.7% (±15.0) for the right and left ears, respectively. These variations likely reflect linguistic differences across populations and reinforce the importance of establishing normative values specific to Brazilian Portuguese^(21,47)^.

For the DDT, ear-specific reference values were proposed for the RE (18–49 vs. 50–59 years) and the LE (18–39 vs. 40–59 years). This asymmetry likely reflects the neurocognitive and neurophysiological demands of dichotic listening, including hemispheric specialization and interhemispheric transfer. In Brazil, reference values of > 95% correct responses have been suggested for adults up to 60 years of age, and > 78% for older adults^(32)^. In the present study, performance in young adults exceeded 95%, whereas scores in individuals aged 50–59 years fell below this threshold, supporting a stricter criterion for younger adults and a more permissive criterion for older adults. For English-speaking adults, a cutoff of 90% has been suggested for individuals aged 18 to 30 years^(48)^. Among Spanish-speaking adults aged 18 to 50 years, mean scores of 98.38% (±3.7%) and a 90th percentile of 96.38% have been reported^(35)^.

Two DDT-related issues warrant emphasis. First, the present findings show that DDT performance varies by both age and ear, consistent with pre-senescent change and with the lateralized mechanisms that support dichotic integration. Second, a potential ceiling effect should be considered, whereby many individuals achieve near-maximal scores, compressing variability and reducing sensitivity to subtle group differences^(35)^. This ceiling effect limits dispersion in the upper performance range and constrains the test’s ability to differentiate among high-performing individuals.

The present study prioritized comparison with national research, given the acoustic and linguistic specificities of Brazilian Portuguese that influence sound pattern perception^(4,7)^. Due to the limited availability of national studies focusing on adults, international investigations were also considered, with appropriate caution in comparative interpretation. All cited studies evaluated healthy individuals without pathological conditions, including hearing loss.

Based on the results presented here, and considering age range and educational level, the adoption of the 10th to 90th percentiles is recommended for interpreting typical performance on the analyzed tests, as follows: MLD, 8–14 dB (18–59 years); GIN, ≤ 6 ms (18–39 years) and ≤ 8 ms (40–59 years); PPS, ≥ 76.6% (18–39 years) and ≥ 63.0% (40–59 years); DPS, ≥ 90.3% (18–29 years), ≥ 80.0% (30–49 years), and ≥ 70.0% (50–59 years); DDT–RE, ≥ 98.5% (18–49 years) and ≥ 93.2% (50–59 years); and DDT–LE, ≥ 97.5% (18–39 years) and ≥ 95.0% (40–59 years). The use of the 5th to 95th percentiles or the mean plus two standard deviations, as presented in Table 3, may be considered for populations with different educational profiles.

Further studies are needed to propose reference values that account for the diversity of the Brazilian population, as well as factors such as musical training and bilingualism.

## CONCLUSION

The results of the AP tests demonstrated significant age-related differences during adulthood, even prior to the onset of senescence. These findings support the proposal of age-specific reference values. Performance variations were heterogeneous across tests over time, reinforcing the need for age-adjusted interpretation of AP test outcomes in adults.
