# Supplementary material for: Behavioral tests of auditory processing: a study on reference values for normal-hearing adults
Source: Codas. 2026 Apr 2;38(3):e20250151. doi: 10.1590/2317-1782/e20250151en (PMC13142685; doi:10.1590/2317-1782/e20250151en)
Supplement: Translation

# Translation: Testes comportamentais do processamento auditivo: um estudo sobre valores de referência para adultos normo-ouvintes

**DOI:** 10.1590/2317-1782/e20250151pt

**Authors:** Sthella Zanchetta, Laura Caetano Meneghelli, Pamela Papile Lunardelo

**Affiliations:** 1 Departamento de Ciências da Saúde, Faculdade de Medicina de Ribeirão Preto, Universidade de São Paulo – FMRP-USP - Ribeirão Preto (SP), Brasil.; 2 Faculdade de Medicina de Ribeirão Preto, Universidade de São Paulo – FMRP-USP - Ribeirão Preto (SP), Brasil.; 3 Departamento de Formação Específica em Fonoaudiologia, Universidade Federal Fluminense – UFF - Nova Friburgo (RJ), Brasil.

**Keywords:** Percepção Auditiva, Testes Auditivos, Adultos, Transtornos da Audição, Audiologia

## Abstract

**Objetivo:**

Analisar e comparar os resultados dos testes clínicos para avaliação do processamento auditivo (PA) em adultos normo-ouvintes, em função do aumento da idade, e propor valores de referência.

**Método:**

Foram avaliados 116 adultos entre 18 a 59 anos, normo-ouvintes e sem queixas auditivas, todos falantes do português brasileiro como língua materna. Os testes utilizados foram: Masking Level Difference (MLD); Gaps-In-Noise (GIN), Padrão de Frequência (TPF) e Padrão de Duração (TPD); Dicótico de Dígitos (TDD).

**Resultados:**

Os resultados do GIN, TPF e TDD apontaram diferenças significativas a partir dos 40 anos; para o TPD a diferença ocorreu entre 18- 29 anos em relação a 50 a 59 anos; para o TDD orelha direita, a diferença foi a partir dos 50 anos e para orelha esquerda aos 40 anos; MLD foi o único teste em que não foi observada diferença em seus resultados com o aumento da idade.

**Conclusão:**

Houve um declínio no desempenho dos testes com o avanço da idade, anterior à senescência. Constataram-se diferenças nos resultados entre os testes, com necessidade de proposição de valores específicos em função das décadas de vida, com exceção do MLD. Os valores propostos são: MLD, 8-14 dB (18-59 anos); GIN, ≤ 6 ms (18-39 anos) ≤ 8 ms (40-59 anos); TPF ≥ 76,6% (18-39 anos), ≥ 63,0% (40-59 anos); TPD, ≥ 90,3% (18-29 anos), ≥ 80,0% (30-49 anos), ≥ 70,0% (50-59 anos); TDD-OD, ≥ 98,5% (18-49 anos), ≥ 93,2% (50-59 anos); TDD-OE, ≥ 97,5% (18-39 anos), ≥ 95,0% (40-59 anos).

## INTRODUÇÃO

Há consenso que o Transtorno do Processamento Auditivo (TPA) pode ser definido como um prejuízo na percepção e no processamento da informação auditiva, verbal e não verbal, que ocorre no sistema nervoso auditivo central (SNAC), acometendo crianças, adultos e pessoas idosas^(1-3)^.

Entidades científicas descrevem quais são as indicações à avaliação do processamento auditivo (PA) na população adulta, elas incluem dificuldades em executar tarefas auditivas relacionadas ao trabalho ou lazer (e.g., música), aprender um novo idioma ou se tornar proficiente nele, afecções neurológicas de qualquer natureza, declínio cognitivo, massas tumorais ou, simplesmente, uma queixa "desproporcional" de dificuldades auditivas em ambientes ruidosos^(1,4-5)^. Outra indicação específica está relacionada às pessoas que chegam à idade adulta já com TPA, como nos casos de transtornos do desenvolvimento (e.g. transtorno do espectro autista)^(2,6)^.

A avaliação para identificação do TPA deve ocorrer por meio de um conjunto de testes comportamentais que contemplem estímulos verbais e não verbais^(1-3)^. Embora não exista uma recomendação sobre uma “bateria mínima” para essa avaliação, há um consenso que os testes escolhidos devem avaliar os diferentes mecanismos, ou categorias, que refletem habilidades auditivas, como a percepção de fala em baixa redundância, escuta dicótica verbal, mecanismos temporais (resolução, ordenação e mascaramento), interação binaural e localização espacial^(7-9)^.

Apesar desses conhecimentos, estudos destinados ao TPA em adultos são escassos. Uma revisão sistemática mostrou que alguns desses trabalhos buscaram caracterizar determinado mecanismo ou habilidade auditiva, em determinada condição de agravo à saúde (e.g. diabetes), e não o diagnóstico do TPA^(10)^.

A vida adulta contempla a faixa etária de 18 a 59 anos, e há evidências de mudanças de desempenho nos testes auditivos comportamentais com o aumento dos anos nesse ciclo^(11-21)^. Um estudo com os testes de resolução temporal^(15)^ demonstrou que adultos com mais de 31 anos apresentaram diferenças de desempenho no teste Gaps-In-Noise (GIN), em relação aos mais jovens; enquanto para o Random Gap Detection Test (RGDT) a diferença significativa foi observada a partir dos 41 anos. Outro estudo^(21)^ também registrou um declínio no desempenho de testes verbais e não verbais, que compõem a avaliação clínica do PA ao longo da vida adulta. Seus autores documentaram, por exemplo, que adultos acima de 40 anos apresentaram resultados inferiores em relação aos de 18-39 anos nos testes Dicótico de Dígitos (TDD) e GIN, já para o Teste Padrão de Duração (TPD) a diferença significativa ocorreu a partir dos 50 anos. Para o teste de escuta dicótica com sentenças^(14)^ a diferença ocorreu entre adultos de 20 a 29 anos em relação a 30 a 39 anos. Essas mudanças significativas nos resultados nos testes auditivos, durante a vida adulta, em sujeitos “eutróficos”, ou seja, na ausência de morbidades, foram denominadas como “declínio saudável” e revelariam as mudanças “naturais” do SNAC^(21)^.

As mudanças de desempenho com o aumento da idade, ainda no período pré-senescência, explicita o desafio da identificação do TPA neste ciclo de vida pela ausência de valores de normalidade. Existem propostas de valores de referência para alguns testes, e esses estudos trazem contribuições significativas na avaliação do PAC^(14,15,19,22-25)^. Contudo, ainda há necessidade de informações complementares, principalmente na identificação em qual idade ocorre a mudança de desempenho. Um estudo^(19)^ avaliou adultos entre 18 e 58 anos em alguns testes do PA, eles foram divididos em duas faixas etárias: 18 a 29 anos e 30 a 58 anos. Essa segunda faixa etária é ampla e contempla idades em que já foram relatadas diferenças significativas de desempenho para alguns testes^(14,15,21)^. Já outros autores, estudaram uma faixa etária mais abrangente, mas o fizeram apenas para os testes GIN e RGDT^(15)^, ou propuseram valores apenas para idade de até 35 anos^(24)^.

A partir dos pressupostos da documentação de mudanças de desempenho em adultos, da preconização da utilização de um conjunto de testes para identificação do TPA e da existência de lacunas quanto a valores de referência ao longo das décadas da vida adulta para os testes de PA, o presente estudo foi desenvolvido com a hipótese de que haverá valores distintos nos resultados dos testes em função do aumento da idade. Para explorar a hipótese, avaliamos adultos, normo-ouvintes entre 18 e 59 anos, em diferentes testes do PA com o objetivo de comparar o desempenho em relação ao aumento da idade e propor valores de referência, para assim contribuir para a avaliação diagnóstica do TPA.

## MÉTODO

Estudo observacional e transversal aprovado pelo Comitê de Ética em Pesquisa da Instituição nº 2.816.793. e 4.826.213. Todos os voluntários que participaram do estudo assinaram o Termo de Consentimento Livre e Esclarecido.

### Participantes

Recrutamento **–** Foram elegíveis sujeitos entre 18 a 59 anos, de ambos os sexos, sem perda auditiva e com boa condição geral de saúde. A amostragem foi por conveniência, recrutada a partir do contato pessoal das pesquisadoras, divulgação por meio de mídias sociais da instituição de ensino onde foi desenvolvida a pesquisa, programas de rádio e televisão de abrangência regional. A divulgação para o recrutamento foi “*Você que não tem queixa auditiva e tem boa condição de saúde, que tal fazer uma avaliação da sua audição*?”, o recrutamento e a avaliação ocorreram em 2019 e entre 2021 e 2025.

No primeiro contato com a pesquisadora por telefone, mensagem telefônica ou e-mail, foram realizados alguns questionamentos para corroborar os aspectos de elegibilidade acima, e identificação de outros critérios de inclusão, a saber: a) ausência de perda auditiva, identificada por meio de exame anterior ou autorreferida; b) ensino médio completo; c) a “boa condição de saúde geral” contemplou a ausência de doenças de origem metabólica, autoimune e neurológica, assim como, suas intercorrências no passado, como como acidente vascular cerebral e traumatismo crânio encefálico; d) ausência de antecedentes de cirurgia otológica; e) exposição ocupacional a níveis elevados de pressão sonora. Os critérios de exclusão, averiguados no dia da coleta, foram a identificação de perda auditiva, de qualquer natureza, em pelo menos uma das orelhas e escore alterado no mini-exame de estado mental.

Compareceram 122 sujeitos, seis foram excluídos, um (0,8%) pelo escore do mini-exame de estado mental e cinco (4,1%) pela identificação de perda auditiva. Os seis sujeitos receberam devolutiva das avaliações e foram encaminhados para avaliação e conduta especializada. Os testes de PA foram realizados em 116 sujeitos.

### Procedimentos

Os testes auditivos foram realizados em cabina acústica, com audiômetro Astera 2 da marca *Interacoustics* e fone HDA 300. As medidas de imitância acústica foram realizadas com sonda de 226 Hz com aparelho Zodiac da marca *Madsen*.

Os procedimentos iniciais foram: a) otoscopia; b) audiometria tonal limiar; c) timpanometria; d) pesquisa do reflexo acústico. A audiometria tonal foi realizada com tom puro, nas frequências de 0,25 a 8 kHz. Foi considerado resultado adequado (ausência de perda auditiva) a média das frequências 0,5, 1, 2 e 4 kHz ≤ 20 dB NA^(26)^ e o limiar de recepção de fala de 0 a 10 dB NS, em relação à respectiva média. A timpanometria e o reflexo acústico foram considerados avaliações complementares; as curvas timpanométricas do tipo A, Ar ou Ad^(27)^, desde que com presença do reflexo acústico nas frequências de 0,5, 1 e 2 kHz na modalidade contralateral, foram consideradas como resultados adequados.

O Mini Exame de Estado Mental foi realizado por um dos autores em todos os sujeitos, apenas aqueles com escore maior ou igual a 29 realizaram os testes de PA^(28)^.

### Testes de processamento auditivo

Todos os testes foram realizados na intensidade de 50 dB NS com referência aos limiares tritonais de fala de cada orelha, ou na intensidade de maior conforto auditivo referido pelo sujeito.

***Masking Level Difference****(MLD)*^(29)^ – O teste é composto por 33 segmentos de ruído de banda estreita que podem ou não conter cinco tons pulsáteis. O teste possui duas variáveis, relação tom-ruído que se alternam nos segmentos e a fase dos tons, SoNo e SπNo. Ao todo sete segmentos sem tom pulsátil, 10 com tom pulsátil em mesma fase com o ruído (SoNo) e 12 com tom em fase oposta ao ruído (SπNo). O resultado foi calculado a partir da subtração do valor relativo em dB da condição SπNo em relação a SoNo.
***Gaps-In-Noise****(GIN)*^(30)^ – O teste é composto por uma série de segmentos de ruído (que varia de 29 a 36, a depender da lista de teste utilizada) com duração de 6 segundos e intervalos entre eles de 5 segundos. Em cada um dos segmentos pode haver até 3 intervalos de silêncio ou nenhum intervalo, quando presentes os intervalos eles podem ter a duração de 2, 3, 4, 5, 6 ,8, 10, 12, 15 e 20 milissegundos. Cada um dos intervalos ocorre seis vezes, em todas as listas, a distribuição dos intervalos nas sequências é randomizada. O teste foi realizado de forma monoaural, com a faixa teste 3 e a ordem de início da testagem entre as orelhas foi alternada entre os participantes. O limiar aproximado (Lap) de resolução temporal foi calculado a partir da recomendação do autor.
**Testes de Ordenação tempora**l – Foram realizados os testes padrão de frequência (TPF) e duração (TPD) versão adulta da Auditec^(31)^, com apresentação binaural. Os resultados foram calculados pela porcentagem de respostas corretas em cada um dos testes.

O TPF é composto por dois tons puros de mesma duração, 200 ms, mas com frequência sonora distinta, um de 880 Hz, denominado “grave” (G) e o outro de 1430 Hz, denominado “agudo” (A). Os tons estão dispostos em 30 sequências, cada uma com três tons, assim cada sequência é composta por dois estímulos de mesma frequência e outro com frequência diferente, que se alternam em seis possibilidades: AAG, AGA, AGG, GGA, GAG e GAA. Os estímulos possuem intervalos intra-estímulos de 150 ms e entre as sequências de 7 seg. A tarefa solicitada foi nomear os três sons pela característica de frequência, na mesma sequência em que ouviu.

O TPD é composto por dois tons puros de mesma frequência, 1.000 Hz, mas com durações distintas, um de 250 ms, denominado “curto” (C) e o outro de 500 ms, denominado “longo” (L). Os tons estão dispostos em 30 sequências, cada uma com três tons, assim cada sequência é composta por dois estímulos de mesma duração e outro com duração diferente, que se alternam em seis possibilidades: CCL, CLL, CLC, LLC, LCC, LCL. Os estímulos possuem intervalos inter-estímulos de 300 ms e entre as sequências de 6 seg. O sujeito foi orientado a nomear os padrões ouvidos, na mesma ordem que ouviu, utilizando o termo “curto” para o som de menor duração e “longo” para o de maior duração.

**Teste dicótico de dígitos** (TDD)^(32)^ – A versão na língua portuguesa do Brasil é composta por 20 sequências, cada uma com quatro números, sendo dois apresentados simultaneamente nas orelhas direita e esquerda e mais outros dois números, também nas orelhas direita e esquerda. Os números que compõem o teste são: quatro, cinco, sete, oito e nove. Esses números são combinados de forma que eles não se repetem na mesma sequência. O teste foi realizado na condição de integração binaural, a aplicação e a análise dos resultados foram realizados conforme preconizado pelo manual^(32)^.

### Análise dos dados

As análises foram conduzidas para caracterização da amostra quanto aos aspectos sociodemográficos e para identificar a existência ou não de relação entre o desempenho nos testes e idade, e, se existente essa relação, buscou-se identificar entre quais faixas etárias os resultados se diferenciavam.

Para o estudo da distribuição dos sexos e dos anos de escolaridade, entre as idades, utilizou-se os testes do *Qui-quadrado* e *Kruskal-Wallis*. O estudo entre os resultados dos testes de PA em relação à variável idade foi realizado em duas direções, na primeira foi investigada a existência ou não de correlação com o teste de correlação de *Spearman.* Na segunda, comparou-se os resultados de cada teste entre as diferentes faixas etárias com o teste de *Kruskal-Wallis* para mais de duas faixas etárias, quando o resultado mostrou diferença significante foi realizado o teste *post hoc* de Dunn, para identificar entre quais ocorreu a diferença, e o teste de *Mann-Whitney* quando a comparação foi entre duas faixas etárias. Esse último teste também foi empregado na comparação dos resultados do teste GIN entre as orelhas. Para o estudo da viabilidade da proposta de valores de referência, de cada teste de PA, foram calculados a média, o desvio padrão e os percentis. O valor de significância foi estabelecido em 5%; o resultado estatisticamente significativo foi assinalado com (*).

## RESULTADOS

Os testes de PA foram realizados em 116 sujeitos, para fins de caracterização em relação às variáveis idade, sexo e anos de escolaridade, eles foram agrupados em quatro faixas etárias distintas (Tabela 1). A distribuição dos sexos e anos de escolaridade não mostrou diferenças estatisticamente significativas entre as faixas etárias (p>0,05). Para a distribuição por sexo da casuística nos testes que contaram com a participação de 20 sujeitos (MLD e GIN), a ocorrência foi de 50,0% (10/20) para cada sexo, em todas as faixas etárias. Quanto à escolaridade da mesma casuística (20 sujeitos), também não foram observadas diferenças estatisticamente significativas entre os grupos (p = 0,0732; 14,3 ± 1,7; 18,1 ± 2,2; 15,8 ± 2,9; 15,3 ± 3,0, para os grupos 1 a 4, respectivamente).

**Tabela 1:** Distribuição e caracterização das variáveis idade, sexo e escolaridade entre as faixas etárias

Variável	Medidas	Faixa etária				*p-value*
		18-29	30-39	40-49	50-59	
		N=30	N=29	N=29	N=28	
Idade	M±dp	22,4±2,6	33,4±2,5	44,6±3,2	53,8±1,3	
Sexo (fem)	n/%	16/53,3	15/51,7	17/58,6	17/60,7	0,8883#
Escolaridade (anos)	M±dp	15,3±2,7	17,1±3,5	16,4±2,7	15,5±4,1	0,0601##
	Min-máx	12-18	12-22	11-23	11-23	

# Teste do Qui-quadrado;

## Teste de Kruskal-Wallis;

**Legenda:** fem = feminino; M = média; dp = desvio padrão

A variação no número de sujeitos entre os testes deve-se à natureza metodológica deste estudo, que integrou dados de duas diferentes coortes transversais conduzidas pelos mesmos pesquisadores, as quais estavam alinhadas quanto aos critérios de inclusão. Cabe destacar que nem todos os testes estiveram presentes em ambas as coortes, em razão dos objetivos específicos de cada uma delas. No entanto, os testes aplicados seguiram protocolos padronizados, assegurando a uniformidade na condução das avaliações.

Os resultados dos testes de PA em cada faixa etária estão na Tabela 2.

**Tabela 2:** Escores dos testes por faixa etária

Testes	Métricas	Faixas etárias			
		18-29	30-39	40-49	50-59
MLD (dB)	n	20	20	20	20
	M ± dp	10,8 ± 1,8	10,8 ± 2,1	10,9 ± 2,1	11,1 ± 2,1
	Mediana	10	10,0	10	12
	Min-máx	6-14	8-16	4-14	8-16
GIN-OD (ms)	n	20	20	20	20
	M ± dp	5,4 ± 0,9	5,0 ± 0,9	6,5 ± 1,1	7,4 ± 1,1
	Mediana	5,5	5,0	6,0	8,0
	Min-máx	4,0-8,0	3,0-6,0	5,0-8,0	5,0-10,0
GIN-OE (ms)	n	20	20	20	20
	M ± dp	5,4 ± 0,9	4,9 ± 0,9	6,5 ± 1,1	7,1 ± 1,2
	Mediana	5,0	5,0	6,0	8,0
	Min-máx	4,0-8,0	3,0-6,0	5,0-8,0	5,0-10,0
TPF (%)	n	30	29	29	28
	M ± dp	94,8 ± 7,8	92,5 ± 9,3	86,6 ± 12,8	83,6 ±13,2
	Mediana	100,0	100,0	86,6	86,6
	Min-máx	73,3-100,0	73,3-100,0	60,0-100,0	46,6-100,0
TPD (%)	n	20	20	20	20
	M ± dp	96,3 ± 6,3	91,9 ± 9,7	90,8 ± 10,15	86,1 ± 12,9
	Mediana	100,0	93,3	93,3	90,0
	Min-máx	73,3-100,0	60,0-100,0	63,3-100,0	53,3-100,0
TDD-OD (%)	n	30	29	29	28
	M ± dp	99,9±0,3	99,4±0,9	98,9±2,0	97,7±2,1
	Mediana	100,0	100,0	100,0	98,7
	Min-máx	98,7-100,0	96,2-100,0	91,2-100,0	88,7-100,0
TDD-OE (%)	n	30	29	29	28
	M ± dp	99,2±1,7	99,45±0,8	98,2±1,9	98,4±1,7
	Mediana	100,0	100,0	98,7	98,7
	Min-máx	98,7-100,0	97,5-100,0	92,5-100,0	93,7-100,0

**Legenda:** ms = milissegundos; M = valor médio; dp = desvio padrão; Min = valor mínimo; Máx = valor máximo; % = porcentagem; n = número de participantes; MLD = Masking Level Difference (sem tradução para o português brasileiro); GIN = Gaps-In-Noise (sem tradução para o português brasileiro); TPF = teste padrão de frequência; TPD = teste padrão de duração; TDD-OD = teste dicótico de dígitos orelha direita; TDD-OE = teste dicótico de dígitos orelha esquerda

Como o teste GIN foi aplicado de forma monoaural a primeira análise foi a comparação dos resultados entre as orelhas, em cada faixa etária, e os resultados mostraram que não houve diferença estatisticamente significante (p>0,05). Os resultados foram: 18-29, p>0,999; 30-39, p=0,5000; 40-49, p>0,9999; 50-59, p=0,2500. Esses resultados permitiram que os resultados do GIN fossem analisados pelo total de orelhas e não de sujeitos.

### Resultados dos testes em função da idade

As análises foram realizadas para verificar a ocorrência ou não de uma correlação significativa entre os resultados dos testes de PA com o aumento da idade, considerando os 116 sujeitos, sem distinção das faixas etárias (Figura 1). Todos os testes, com exceção do MLD (p>0,05), apresentaram correlação estatisticamente significante entre seus resultados e o aumento de idade (p<0,05*). Houve um declínio de desempenho com o aumento da idade, com exceção do MLD.

**Figura 1:** Correlação entre o desempenho nos testes MLD (A), GIN (B), TPF (C), TPD (D) e TDD (E e F) em função da idade

**Legenda:** % = porcentagem; r = coeficiente de correlação de Pearson; p = valor de significância; MLD = Masking Level Difference (sem tradução para o português brasileiro); GIN = Gaps-In-Noise (sem tradução para o português brasileiro); TPF = teste padrão de frequência; TPD = teste padrão de duração; TDD-OD = teste dicótico de dígitos orelha direita; TDD-OE = teste dicótico de dígitos orelha esquerda

### Análise para proposição de valores de referência

Nesse momento, os resultados dos testes de PA foram analisados entre as diferentes faixas etárias. Quando observada diferença significativa, uma segunda análise foi realizada, com o teste de *post hoc*, para identificar entre quais faixas etárias ocorreu essa diferença, para então explorar os dados e verificar a necessidade e viabilidade de valores de referência distintos, em cada teste, ao longo da vida adulta.

MLD – Não foi observada diferença dos limiares (p>0,8352) entre as diferentes idades (Figura 2A). Com esse resultado, entende-se que os limiares do teste MLD podem ser os mesmos para a faixa etária de 18-59, o valor médio, o desvio padrão e os percentis estão apresentados na Tabela 3. Considerando o percentil 10-90, os valores de referência de normalidade estão entre 8 a 14 dB (Tabela 3).

**Figura 2:** Gráfico boxplot representando a distribuição dos desempenhos dos testes MLD (A), GIN (B), TPF (C), TPD (D) e TDD (E e F) nas diferentes faixas etárias. Os limites inferiores e superiores do boxplot representam os percentis 25 e 75, as hastes inferiores e superiores os percentis 10 e 90, e a linha interna a mediana

**Legenda:** % = porcentagem; MLD = Masking Level Difference (sem tradução para o português brasileiro); GIN = Gaps-In-Noise (sem tradução para o português brasileiro); TPF = teste padrão de frequência; TPD = teste padrão de duração; TDD-OD = teste dicótico de dígitos orelha direita; TDD-OE = teste dicótico de dígitos orelha esquerda

**Tabela 3:** Métricas e proposição de escores de desempenho dos testes MLD, GIN, TPF, TPD e TDD para idade adulta

Testes	Idade	n	Métricas						
			M ± dp	Percentis					
				P5	**P10**	P25	P75	**P90**	P95
MLD (dB)									
	18-59	80	10,9 ± 2,0	8	**8**	10	12	14,0	14
GIN (ms)									
	18 – 39	80	5,1 ± 0,8	4	4	5	6	**6**	6
	40 – 59	80	6,5 ± 1,1	5	5	6	8	**8**	8
TPF (%)									
	18 – 39	59	93,7 ± 8,6	76,0	**76,0**	90,0	100,0	**100,0**	100,0
	40 – 59	57	84,7 ± 12,9	60,0	**63,0**	76,6	96,6	**100,0**	100,0
TPD (%)									
	18 – 29	20	96,3 ± 6,3	74,1	**90,3**	93,3	100,0	**100,0**	100,0
	30 – 49	40	91,4 ± 9,5	63,8	**80,0**	86,6	100,0	**100,0**	100,0
	50 – 59	20	86,1 +/- 12,9	54,1	**70,0**	74,9	96,6	**100,0**	100,0
TDD-OD (%)									
	18-49	88	99,4 ± 0,8	97,5	**98,5**	100,0	100,0	**100,0**	100,0
	50 – 59	28	97,6 ± 2,9	88,7	**93,2**	96,5	100,0	**100,0**	100,0
TDD-OE (%)									
	18 – 39	59	99,4 ± 0,9	97,5	**97,5**	98,5	100,0	**100,0**	100,0
	40 – 59	57	97,8 ± 1,9	94,3	**95,0**	96,5	100,0	**100,0**	100,0

**Legenda:** % = porcentagem; dp = desvio padrão; n = número de participantes; MLD = Masking Level Difference (sem tradução para o português brasileiro); GIN = Gaps-In-Noise (sem tradução para o português brasileiro); TPF = teste padrão de frequência; TPD = teste padrão de duração; TDD-OD = teste dicótico de dígitos orelha direita; TDD-OE = teste dicótico de dígitos orelha esquerda

GIN – A comparação dos valores de Lap entre os quatro grupos mostrou diferença estatisticamente significante (p < 0,0001*). O teste *post hoc* identificou que as diferenças ocorreram entre o grupo de 18–29 anos em relação aos grupos de 40–49 (p = 0,0001*) e 50–59 anos (p < 0,0001*), e entre o grupo de 30–39 anos em relação aos grupos de 40–49 (p < 0,0001*) e 50–59 anos (p < 0,0001*). Não foram observadas diferenças significativas entre os grupos de 18–29 e 30–39 anos (p > 0,9999), nem entre os grupos de 40–49 e 50–59 anos (p = 0,2176) (Figura 2B). Os grupos de maior idade apresentaram valores de Lap mais elevados em comparação aos mais jovens, indicando pior desempenho.

A partir dessas análises, os resultados foram agrupados em duas faixas etárias: 18–39 e 40–59 anos. Foi conduzida, novamente, uma análise comparativa dos Lap entre essas idades, com resultado estatisticamente significante (p < 0,0001*). As medidas descritivas de valor médio, desvio padrão e percentis estão apresentadas na Tabela 3. Com base no percentil 90, recomenda-se o valor de Lap de até 6 ms como referência de normalidade para a faixa etária de 18–39 anos, e de até 8 ms para 40–59 anos (Tabela 3).

TPF – A análise comparativa dos acertos no TPF entre as quatro faixas etárias evidenciou uma diferença estatisticamente significante *(p* = 0,0005*). O teste de *post hoc* identificou que as diferenças ocorreram entre as idades de 18–29 anos em relação a 40–49 (p = 0,0273*) e 50–59 anos (p = 0,0015*), e entre 30–39 anos em relação a 50–59 anos (p = 0,0240*). Não foram observadas diferenças significativas entre os demais pares (18–29 vs. 30–39, p > 0,9999; 30–39 vs. 40–49, p > 0,2401; 40–49 vs. 50–59, p > 0,9999) (Figura 2C). Mais uma vez, os sujeitos de maior idade apresentaram pior desempenho em comparação aos mais jovens.

A partir dessas análises, os resultados foram agrupados em duas faixas etárias: 18–39 e 40–59 anos. Foi conduzida, novamente, uma análise comparativa dos resultados do TPF entre essas duas faixas etárias, com resultado estatisticamente significante (p < 0,0001*). As medidas descritivas de valor médio, desvio padrão e percentis estão apresentadas na Tabela 3. Com base no percentil 10, recomendam-se escore maior ou igual a 76,6% como valor de referência de normalidade para a faixa etária de 18–39 anos, e maior ou igual a 63,3% para 40–59 anos.

TPD – Houve diferença estatisticamente significante nos resultados do TPD entre as faixas etárias (p = 0,0196*). O teste *post hoc* indicou que a diferença ocorreu apenas entre 18–29 anos em relação à de 50–59 anos (p = 0,0112*), não havendo diferenças significativas entre os demais pares (18–29 vs. 30–39, p = 0,5622; 30–39 vs. 40–49, p > 0,9999; 30–39 vs. 50–59, p = 0,9082; 40–49 vs. 50–59, p > 0,9999) (Figura 2D). Esses resultados sugerem que apenas os sujeitos de menor e maior idade apresentaram desempenhos distintos, sendo o de maior idade com desempenho inferior (Tabela 3). Com base nesses achados, definiu-se que os escores de referência deveriam considerar três faixas etárias distintas: 18–29, 30–49 e 50–59 anos. A análise comparativa entre essas três faixas etárias revelou, novamente, uma diferença significativa entre os grupos (p = 0,0074*), sendo a única diferença observada entre os grupos de menor e maior idade (p = 0,0056*), sem diferenças significativas entre os demais (p > 0,05). As medidas descritivas de valor médio, desvio padrão e percentis estão apresentadas na Tabela 3. Assim, os valores de referência de normalidade propostos, com base no percentil 10, são escores maiores ou iguais a 90,3% para 18–29 anos, 80,0% para 30–49 anos e 70,0% para 50–59 anos (Tabela 3).

TDD – Os escores das orelhas direita (OD) e esquerda (OE) foram analisados separadamente. Houve diferença estatisticamente significante nas porcentagens de acertos entre os grupos (OD, p<0,0001*; OE, p=0,0097*).

Para os acertos da OD, o teste *post hoc* mostrou que a diferença ocorreu entre as faixas etárias de 18–29, 30–39 e 40–49 anos em relação à de 50–59 anos (respectivamente, p < 0,0001*; p = 0,0029*; p = 0,0365*), sendo a faixa etária de 50–59 anos aquela com escores inferiores às outras (Figura 2E). As demais comparações entre os grupos não foram estatisticamente significantes (18–29 vs. 30–39, p = 0,6770; 18–29 vs. 40–49, p = 0,1131; 30–39 vs. 40–49, p > 0,999).

A partir desses resultados, decidiu-se estudar os valores de referência considerando duas faixas etárias: 18–39 e 40–59 anos. A análise comparativa dos acertos entre essas duas faixas etárias revelou uma diferença estatisticamente significante (p < 0,0001*). As medidas descritivas de valor médio, desvio padrão e percentis estão apresentadas na Tabela 3. Com base no percentil 10, os valores de referência de normalidade propostos são escores iguais ou maiores a 98,5% para a faixa de 18–39 anos e 93,2% para 40–59 anos (Tabela 3).

A análise dos acertos no TDD-OE entre as quatro faixas etárias mostrou diferença estatisticamente significante (p = 0,0010*). O teste *post hoc* revelou diferenças significativas entre os grupos de 18–29 anos em relação aos de 40–49 e 50–59 anos (respectivamente, p = 0,0499* e p = 0,0196*), bem como entre os grupos de 30–39 anos em relação aos de 40–49 e 50–59 anos (respectivamente, p = 0,0442* e p = 0,0173*) (Figura 2F). Não houve diferenças significativas entre os grupos de 18–29 e 30–39 anos (p > 0,9999), nem entre 40–49 e 50–59 anos (p > 0,9999).

Os resultados do TDD-OE também foram agrupados em duas faixas etárias 18-39 e 40-59, e na nova análise comparativa dos resultados entre elas mostrou diferença significativa (p=0,0063*). As medidas descritivas do valor médio, desvio padrão e dos percentis estão apresentadas na Tabela 3. A partir do percentil 10, recomenda-se os escores maiores ou iguais a 97,5% para a idade entre 18-39 e maiores ou iguais a 95,0% para 40–59 anos , como valores de referência de normalidade.

## DISCUSSÃO

O presente estudo teve como objetivo investigar e comparar os resultados dos testes MLD, GIN, TPF, TPD e TDD em adultos normo-ouvintes em função do avanço da idade, bem como propor valores de referência.

O recrutamento dos sujeitos buscou garantir a representatividade da comunidade. A integridade da sensibilidade auditiva, as ausências de comprometimento do estado de consciência mental e diferenças do nível de escolaridade eliminaram fatores que possuem influência nos resultados desses testes^(21)^. Essa tomada de decisão permitiu que as diferenças de desempenho encontradas em nosso estudo possam ser atribuídas à variável idade, diminuindo a ocorrência de vieses que poderiam se sobrepor aos resultados.

O uso do percentil para definição dos valores de referência merece ser discutido. Essa métrica é utilizada na interpretação dos testes de PA^(14,33-36)^; apesar do valor médio das respostas acrescido de dois desvios padrão seja a mais frequentemente recomendada^(1-3,37-39)^.

Em nossa pesquisa, os resultados dos testes auditivos não apresentaram distribuição normal, ou seja, a média de acertos não representa adequadamente a resposta mais frequente dos sujeitos. Nesse caso, o cálculo da dispersão se torna mais difícil, ou seja, o quanto a resposta de um sujeito varia em função das respostas mais frequentes, assim o desvio ou erro padrão não retrata essa variabilidade, onde o percentil é a escolha recomendada^(40)^.

O percentil permite uma distribuição ordenada das respostas, indo além da categorização binária (normal vs. alterado), pois posiciona o indivíduo em relação ao seu grupo de referência^(40)^. O uso das duas medidas, valor médio com desvio padrão e percentis, já foi utilizado para propor valores de referência para um teste de PA, nesse estudo os autores ao analisarem as duas medidas observaram diferença mínima entre elas, por esse motivo recomendaram o uso da medida central^(41)^. Outros estudos que propuseram valores de referência para testes de PA também realizaram mais de uma métrica para descrever seus resultados, como a média com dois desvios padrão e o percentil^(35,36)^. Em nosso estudo, com exceção do TPD, os valores dos percentis 10 e 90 para os testes MLD, GIN, TPF e TDD também foram bastante próximos dos valores médios acrescidos de dois desvios padrão. Contudo, os valores de referência aqui propostos foram baseados nos percentis 10 e 90, outros percentis foram incluídos com objetivo de proporcionar uma caracterização mais precisa dos resultados, seja na avaliação ou reavaliação pós-treinamento auditivo^(40)^.

### Valores de referência dos testes de PA

As alterações estruturais e fisiológicas do SNAC na vida adulta influenciaram significativamente o desempenho nos testes clínicos de PA, com exceção do MLD. Essas alterações podem ser atribuídas a mudanças neurofisiológicas e nos neurotransmissores do sistema nervoso central^(11,20,21,42)^. Observou-se que a idade em que essas mudanças se manifestaram variou entre os testes e, em alguns casos, até entre as orelhas, como no TDD — resultado esperado, dado que as mudanças fisiológicas e bioquímicas ocorrem em momentos distintos, dependendo da região cerebral^(11,20)^.

O MLD foi o único teste sem variações significativas de resultado entre as faixas etárias, permitindo um valor de limiar único para adultos de 18 a 59 anos. O MLD investiga o mecanismo de interação binaural, relacionado à função auditiva do VIII par craniano e tronco encefálico, ou seja, região subcortical^(43)^. Assim, a ausência de diferença entre faixas etárias nesse ciclo de vida pode ser atribuída ao fato de que até os 60 anos as alterações ocorrem predominantemente em áreas corticais. Os limiares entre 8 e 14 dB, com base nos percentis 10 e 90, são próximos aos já relatados de — 10,2 a 11,4 dB em mulheres de 20 a 30 anos^(25)^. No entanto, são inferiores aos 17–18 dB sugeridos por outro estudo^(19)^, com sujeitos de 18–58 anos.

No teste GIN, não houve diferença de respostas entre orelhas em nenhuma faixa etária, resultado já documentado^(15,23)^. O declínio dos Lap a partir dos 40 anos já foi relatado em um estudo que avaliaram 176 sujeitos de 20 a 85 anos, seus autores atribuíram esse achado às alterações do SNAC^(13)^. Contudo, outro estudo^(44)^ não encontrou diferenças nos Lap entre jovens e adultos de meia-idade, porém foi constatada uma menor porcentagem de identificação dos gaps no grupo de maior idade, o que foi associado à redução da habilidade temporal em mulheres de meia idade^(44)^.

Os valores de referência para Lap no GIN, com base no percentil 90, foram de 6 ms (18–39 anos) e 8 ms (40–59 anos) — idênticos aos já sugeridos anteriormente^(15)^. Esses valores também se assemelham aos propostos por outros estudos, aos 4,19 ms, para adultos jovens^(23)^, 6 ms para adultos com idade média de 39 anos^(36)^ e 6,6 ms (±1,3) para meia-idade^(45)^.

Para o TPF, os valores de referência a partir do percentil 10 foram de 76,6% (18–39 anos) e 63,3% (40–59 anos). Em outro estudo, os autores sugeriram valores ≥86,6% para 18–29 e 30–58 anos^(19)^, considerando já o acréscimo de dois desvios padrão. Em falantes de espanhol, foram propostos valores ≥71,4% para adultos de 18 a 50 anos, com idade média de 29 anos^(35)^, valor mais próximo ao aqui proposto^(35)^.

Outros estudos usaram outra versão de TPF^(46)^, que difere na frequência sonora (1.122 Hz vs. 1.430 Hz), na duração dos tons (150 ms vs. 200 ms) e no intervalo entre os tons (200 ms vs. 15 ms), da versão adotada no presente estudo. Em um estudo com 28 adultos holandeses entre 18 e 47 anos (média de idade de 29 anos), foi relatado como referência, a partir do percentil 10, o escore de 89% de acertos^(33)^. Em outra pesquisa, realizada com 76 adultos poloneses entre 18 e 54 anos (média de idade de 39 anos), também com base no percentil 10, foi proposto o escore de 56,7%^(36)^. Essas diferenças entre estudos indicam que os valores de referência para o TPF não são homogêneos, mesmo com a mesma versão do teste. Fatores como amostras pequenas, faixas etárias amplas e idioma nativo podem explicar tais discrepâncias. O idioma, especialmente, é um fator relevante nos testes de ordenação temporal^(47)^.

Para o TPD, foram estabelecidas três faixas etárias com valores normativos distintos. O ponto de corte de 90,3% (percentil 10) para 18–29 anos é superior ao de 73,3% encontrado em estudo anterior^(19)^. Para 30–49 anos, o escore foi de 80%, também acima do valor de 70% proposto para essa faixa etária^(19)^. Outro estudo apresentou 83,3% (percentil 3) para jovens adultos brasileiros, utilizando outra versão do TPD^(46)^, cuja única diferença em relação à versão aqui utilizada é o tempo entre sequências (7 vs. 6 segundos)^(22)^. Nesse mesmo estudo, foi ainda reportado o valor do percentil 25, 93,3% de acertos, coincidente com o do presente estudo, considerando o mesmo percentil. Em pesquisas com falantes de outros idiomas, os resultados são variados. Em adultos poloneses entre 18 e 54 anos (idade média de 39 anos), foi proposto o valor de 55,3% (percentil 10)^(36)^. A versão aqui utilizada possui a recomendação do escore de 67% como valor normativo^(31)^, para a outra versão disponível do teste, o seu autor propôs o valor de 73% (percentil 10)^(46)^, enquanto outros trabalhos relataram escores médios de 86,5% (±13,0) e 79,7% (±15,0) para orelhas direita e esquerda, respectivamente. Essas variações refletem possíveis diferenças linguísticas entre populações, reforçando a importância de valores normativos específicos para o português brasileiro^(21,47)^.

No TDD, foram propostos valores distintos entre orelhas e faixas etárias: TDD-OD (18–49 e 50–59 anos) e TDD-OE (18–39 e 40–59 anos). Essas diferenças refletem mecanismos distintos da escuta dicótica. No Brasil, foram sugeridos valores ≥95% de acertos para adultos até 60 anos e >78% para idosos^(32)^. Em nosso estudo, os valores para adultos jovens foram superiores a 95,0%, enquanto para adultos entre 50–59 anos, ficaram abaixo desse valor, sugerindo um padrão de normalidade mais rigoroso para jovens e menos rígido para adultos mais velhos. Em relação a falantes de inglês, foi sugerido 90% como ponto de corte para indivíduos de 18 a 30 anos^(48)^. Em adultos espanhóis (18–50 anos), foram encontradas médias de 98,38% (±3,7%), com percentil 90 de 96,38%^(35)^.

Dois aspectos sobre o TDD devem ser destacados. O primeiro deles é que a partir dos resultados do presente estudo, observamos que ocorreram modificações no TDD em função do aumento da idade e da orelha, refletindo não apenas o processo natural de pré-senescência, mas também aspectos da escuta dicótica. O segundo é o possível efeito teto, quando muitos sujeitos atingem o desempenho máximo, reduzindo a variabilidade e a sensibilidade do teste para detectar diferenças sutis^(35)^. Esse efeito reduz a variabilidade dos dados, limitando a capacidade do teste de identificar diferenças sutis entre indivíduos de alto desempenho.

Este estudo priorizou a discussão com pesquisas nacionais, considerando as especificidades acústicas do idioma, que afetam a percepção de padrões sonoros^(47)^. Devido à escassez de literatura nacional para adultos, estudos internacionais também foram incluídos, com as devidas ressalvas comparativas. Todos os estudos analisaram sujeitos hígidos e sem condições patológicas, incluindo perda auditiva.

A partir dos resultados aqui apresentados, considerando as faixas etárias e o nível de escolaridade sugere-se adotar o percentil 10-90 para interpretação dos resultados adequados aos testes estudados, a saber: MLD, 8-14 dB (18-59 anos); GIN, ≤ 6 ms (18-39 anos) ≤ 8 ms (40-59 anos); TPF ≥ 76,6% (18-39 anos), ≥ 63,0% (40-59 anos); TPD, ≥ 90,3% (18-29 anos), ≥ 80,0% (30-49 anos), ≥ 70,0% (50-59 anos); TDD-OD, ≥ 98,5% (18-49 anos), ≥ 93,2% (50-59 anos); TDD-OE, ≥ 97,5% (18-39 anos), ≥ 95,0% (40-59 anos). A utilização do percentil 5–95 ou da média acrescida de dois desvios-padrão, conforme apresentado na Tabela 3, pode ser considerada uma alternativa em situações com diferentes perfis de escolaridade. Há necessidade de mais trabalhos com proposição de valores de referência considerando a diversidade da população brasileira, assim como os aspectos da experiência musical e do bilinguismo.

## CONCLUSÃO

Os resultados dos testes de PA mostraram diferenças significativas em função da idade, mesmo ainda na fase adulta, anterior à senescência. Isso justificou a proposição de valores de referência distintos conforme a faixa etária. As variações no desempenho manifestaram-se de forma heterogênea em relação aos testes, ao longo do tempo.
